# Efficient Exciton Dislocation and Ultrafast Charge Extraction in CsPbI_3_ Perovskite Quantum Dots by Using Fullerene Derivative as Semiconductor Ligand

**DOI:** 10.3390/nano12183101

**Published:** 2022-09-07

**Authors:** Yusheng Li, Dandan Wang, Shuzi Hayase, Yongge Yang, Chao Ding, Qing Shen

**Affiliations:** Faculty of Informatics and Engineering, The University of Electro-Communications, 1-5-1 Chofugaoka, Chofu, Tokyo 182-8585, Japan

**Keywords:** CsPbI_3_, quantum dot, fullerene derivative, exciton dislocation, extraction

## Abstract

CsPbI_3_ quantum dots (QDs) are of great interest in new-generation photovoltaics (PVs) due to their excellent optoelectronic properties. The long and insulative ligands protect their phase stability and enable superior photoluminescence quantum yield, however, limiting charge transportation and extraction in PV devices. In this work, we use a fullerene derivative with the carboxylic anchor group ([SAM]C60) as the semiconductor ligand and build the type II heterojunction system of CsPbI_3_ QDs and [SAM]C60 molecules. We find their combination enables obvious exciton dislocation and highly efficient photogenerated charge extraction. After the introduction of [SAM]C_60_, the exciton-binding energy of CsPbI_3_ decreases from 30 meV to 7 meV and the fluorescence emission mechanism also exhibits obvious changes. Transient absorption spectroscopy visualizes a ~5 ps electron extraction rate in this system. The findings gained here may guide the development of perovskite QD devices.

## 1. Introduction

Colloidal halide perovskite quantum dots (QDs) have emerged as one of the most attractive materials because of their simple synthesis method, improved stability, flexible compositional control, size-tunable bandgap, unprecedented high photoluminescence quantum efficiency (PLQY), efficient multiple-exciton effects, and slow hot carrier cooling [1,2,3,4]. These excellent properties endow perovskite QDs with great potential for application in photodetectors, solar devices, lasers, and light-emitting diodes [5,6,7,8]. Among all the halide perovskite QDs with ABX_3_ formula (A = CH_3_NH_3_^+^, CH_3_(NH_2_)_2_^+^, and Cs^+^; B = Pb^2+^, Sn^2+^; X = I^−^, Br^−^, and Cl^−^), all-inorganic CsPbI_3_ QDs are by far the most studied because they have higher stability and PLQY than organic–inorganic hybrid parts [2,9,10]. In addition, CsPbI_3_ QDs are widely used as photon-absorber materials in perovskite QD solar cells, and the efficiency of state-of-the-art CsPbI_3_ QD solar cell has reached up to 16.21%, which is much more efficient than other semiconductor QD solar cells [11].

Nevertheless, there is still a huge gap compared with bulk CsPbI_3_ solar cells [12]. Although the size effects and long alkyl chain ligands endow perovskite materials with higher stability and higher defect tolerance, they result in more bound excitons, poorer charge transportation, and more difficult charge extraction [13,14,15]. Using weakly polar anti-solvents, such as methyl acetate, can remove the long alkyl chain ligands during layer-by-layer preparation of QD film, facilitating charge transportation and extraction [16]. In this method, precise control of atmospheric moisture can aid the hydrolysis of methyl acetate to generate acetic acid that replaces native oleate ligands, while high atmospheric moisture results in the yellow phase transition [16]. It is also suggested that both processed time and numbers have a big influence on the QD film quality [17]. Although this method has gained outstanding success, the complicated processes and rigorous conditions make the method difficult to repeat. Another method is directly using short-chain ligands to replace long-chain ligands during synthesis or ligand exchange processes [18]. However, the colloidal solution of perovskite QDs with short ligands usually suffers from aggregation because of the weak steric effect of short ligands [19]. Additionally, the polar of short-chain ligands usually has negative effects on the chemical stability of CsPbI_3_ QD. Currently, finding approximate ligands that can not only facilitate exciton dislocation and extraction, but also have no negative effects on the chemical structure of CsPbI_3_ QD is urgently required for more efficient CsPbI_3_ QD photovoltaics. In recent years, fullerene derivatives, as excellent electron acceptors, have been broadly used in organic solar cells and bulk perovskite solar cells [20,21]. Their combination with perovskite QDs was also revealed to be beneficial for photogenerated carrier separation [22,23]. However, their potential as semiconductor ligands for perovskite QDs and their impacts on the exciton properties have not been fully discussed.

**Scheme 1:**
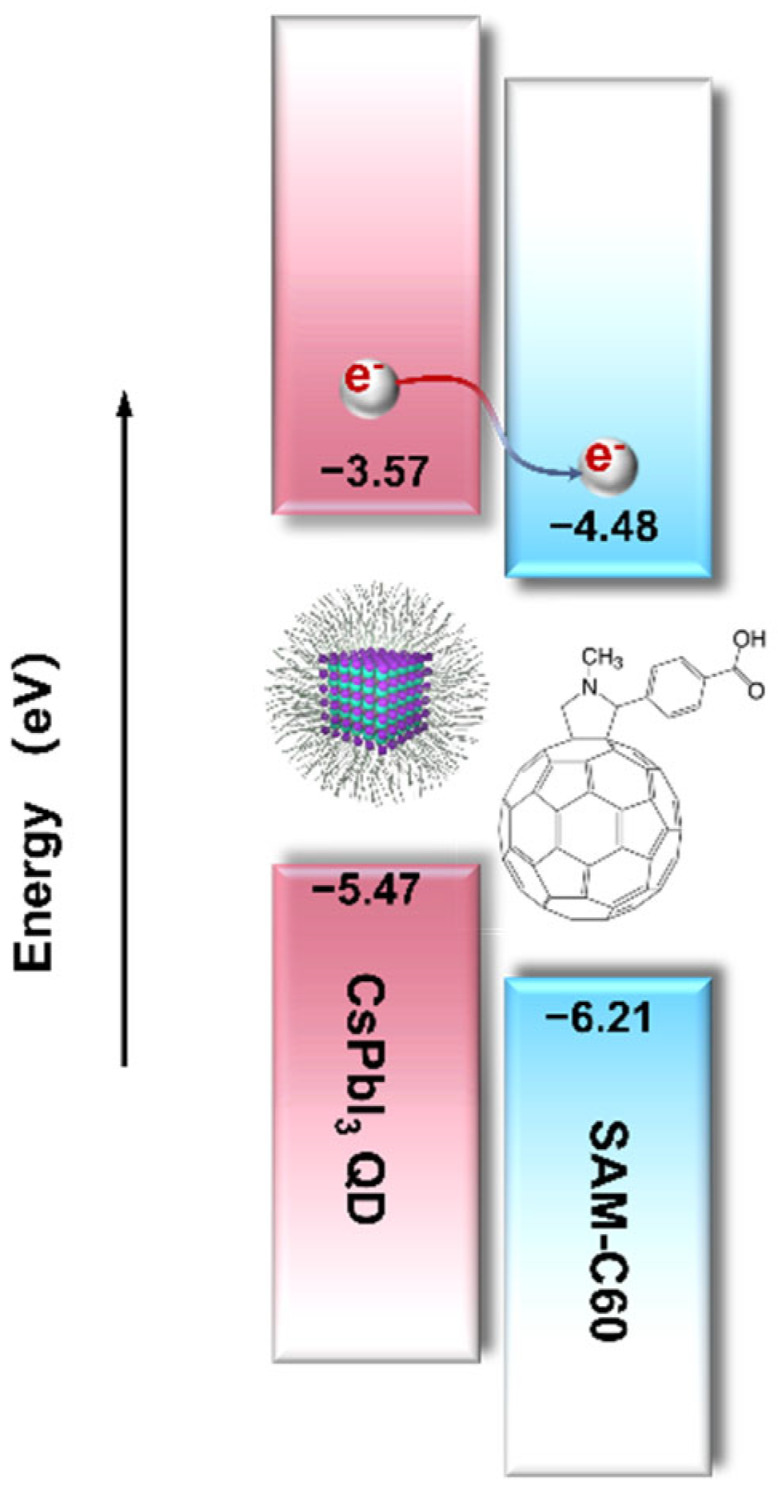
Alignment of the energy levels of CsPbI_3_ QDs and [SAM]C60. The indicated energy values are obtained from refs [24,25] and following absorption spectroscopy.

In this work, we achieve efficient exciton dislocation and extraction in CsPbI_3_ QDs by using a fullerene derivative, 4-(1′,5′-Dihydro-1′-methyl-2′H-[5,6]fullereno-C60-Ih-[1,9-c]pyrrol-2′-yl)benzoic acid (hereafter called [SAM]C60), chosen for its surface-anchoring carboxylic group and its ability to form type II heterojunction system with CsPbI_3_ QDs (Scheme 1). We find the introduction of [SAM]C60 has no negative effects on the chemical structure of CsPbI_3_ QD, but it obviously decreases the exciton-binding energy of CsPbI_3_ QD and changes the fluorescence emission mechanism. Concurrently, ultrafast electron extraction (5 ps) from CsPbI_3_ QDs to [SAM]C60 is directly visualized by transient absorption spectroscopy. The results gained here provide an alluring strategy for changing the transportation properties and achieving efficient carrier extraction in CsPbI_3_ QDs.

## 2. Results and Discussion

**Scheme 2:**
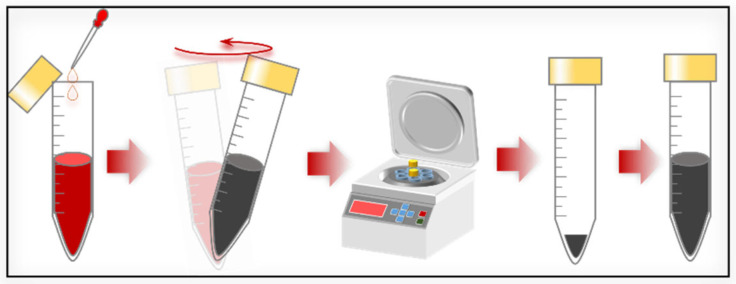
The preparation processes of CsPbI_3_-[SAM]C60 QDs.

**Figure 1:**
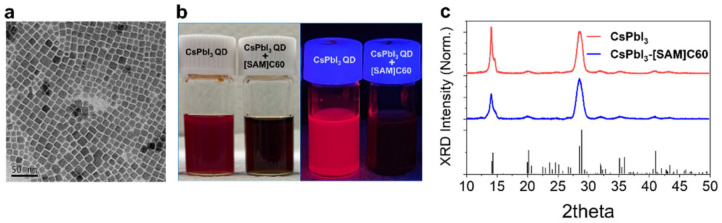
(**a**) Transmission electron microscopy (TEM) image of CsPbI_3_ QDs. (**b**) Digital images of CsPbI_3_ QDs and CsPbI_3_-[SAM]C60 QD dispersions under room light (left) and excitation using an ultraviolet lamp (right) at room temperature, separately. (**c**) XRD of CsPbI_3_ QDs and CsPbI_3_-[SAM]C60 QDs. The black line is the PDF card of γ-CsPbI_3_.

CsPbI_3_ QDs were synthesized using the previously reported hot injection method, with slight modification [1,7]. The as-prepared CsPbI_3_ QDs exhibit an average diameter of 12.4 nm and high crystalline quality, as shown in Figure 1a. The [SAM]C60-adsorbed CsPbI_3_ QDs dispersion (indicated hereafter as CsPbI_3_-[SAM]C60 QDs) was obtained according to the processes in Scheme 2. Firstly, we added tiny [SAM]C60 into CsPbI_3_ QDs hexane dispersion and stirred for 30 seconds. The mixture was then centrifuged and redispersed into toluene. As shown in Figure 1b, CsPbI_3_ QDs dispersion immediately transformed from red to black under room light after the tiny [SAM]C60 molecules were introduced, which means our [SAM]C60 molecules can effectively anchor onto the surface atoms of CsPbI_3_ QDs and form stable CsPbI_3_-[SAM]C60 complexes. In toluene, CsPbI_3_-[SAM]C60 QDs remain very well dispersed, which is attributed to the big steric effect of [SAM]C60 molecules. Moreover, the obvious fluorescence quenching in the complexes under ultraviolet lamp excitation is ascribed by photogenerated electron transfer from CsPbI_3_ QDs to [SAM]C60 molecules. According to the results of XRD in Figure 1c, both CsPbI_3_ QDs and CsPbI_3_-[SAM]C60 crystallized in the orthorhombic phase indicating that [SAM]C60 molecules have no negative effect on the chemical structure of CsPbI_3_ QDs.

**Figure 2:**
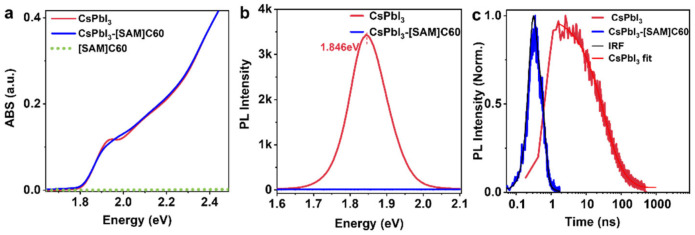
(**a**) Absorption spectra (ABS), (**b**) photoluminescence (PL), (**c**) time of resolved photoluminescence (TRPL) of CsPbI_3_ QDs and CsPbI_3_-[SAM]C60 QDs.

Figure 2a shows the absorption spectra of CsPbI_3_ QDs, CsPbI_3_-[SAM]C60 QDs and [SAM]C60 molecules. We notice that there is a significant difference located around the absorption edge where the exciton peak becomes smoother in CsPbI_3_-[SAM]C60 QDs. Since the absorption of the [SAM]C60 molecules (green dot line) we used can be completely ignored when compared with the intrinsic absorption of CsPbI_3_ QDs, we attribute the absorption difference to the influence of [SAM]C60 molecules on the exciton state of CsPbI_3_ QDs. Evidence of carrier transfer can be found via the photoluminescence (PL) spectrum (Figure 2b,c). The near-zero PLQY of CsPbI_3_-[SAM]C60 QDs, in contrast with the high PLQY from the oleate-passivated QDs (95%), demonstrates the significant carrier extraction ability of our [SAM]C60 (extraction efficiency is near 100%). [SAM]C60 also has a pronounced effect on the PL decay characteristics of the CsPbI_3_ QDs. The time of resolved PL measurements (TRPL) shows an average lifetime of 51.09 ns for CsPbI_3_ QDs. However, for CsPbI_3_-[SAM]C60 QDs, the PL decay is shorter than the instrument response function (IRF, ~100 ps), indicating fast carrier transfer from CsPbI_3_ QDs to [SAM]C60.

**Figure 3:**
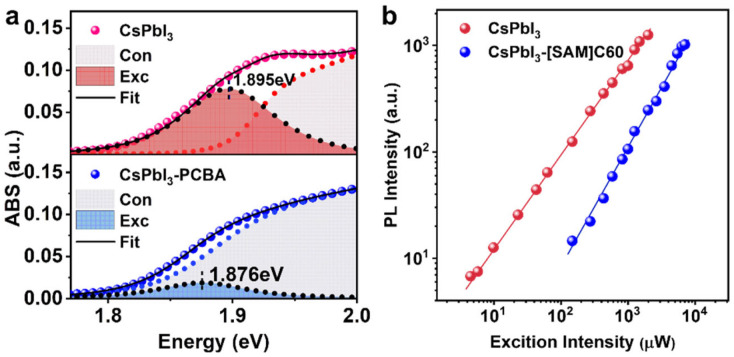
(**a**) The band-edge absorption of CsPbI_3_ QDs and CsPbI_3_-[SAM]C60 QDs fitted with Elliott formula (Con and Exc represent continuous band and exciton absorption, respectively). (**b**) Excitation power-dependent PL spectra.

To further reveal the influence of [SAM]C60 molecules on the exciton state of CsPbI_3_ QDs, we fit the band-edge absorption with the Elliott formula [26,27]:

(1) $$\alpha (h\omega )=\frac{A}{h\omega }\sum_{j}\frac{4\pi \sqrt{E_{b}^{3}}}{j^{3}}\delta \left(h\omega -(E_{g}-\frac{E_{b}}{j^{2}})\right)+\frac{2\pi }{1-e^{-2\pi \sqrt{\frac{E_{b}}{h\omega -E_{g}}}}}\sqrt{E_{b}}\theta (h\omega -E_{g})$$

where *A* is constant, *hω* is the photon energy, *E_b_* is the binding energy of the exciton, *E_g_* is the optical bandgap, and *δ* and *θ* are Delta function and Heaviside functions, separately. The broadening of absorption in a nonideal lattice is simulated by convolving with Gauss functions (broadening width is T). The exciton and continuous band contributions of the band-edge absorption spectrum are separated by fitting the experimental data (see Figure 3a). The fitting parameters are tabulated in Table 1. We notice there is a small redshift (~20 meV) in the bandgap for CsPbI_3_-[SAM]C60 QDs, as well as a decrease in exciton binding energy from 25 meV (CsPbI_3_-[SAM]C60 QDs) to 7 meV (CsPbI_3_ QDs). This result indicates significant exciton dislocation and weakened Coulomb interaction in CsPbI_3_-[SAM]C60 QDs. The same broadening width (T) further supports that [SAM]C60 has no negative impact on CsPbI_3_ QDs. Figure 3b shows that an excitation power-dependent PL spectrum fitted with a power law I_PL_~Lexc^α^, where I_PL_ is the PL intensity, L_exc_ is the power of the exciting laser (wavelength 470 nm, much higher than the bandgap of QDs), and α is the coefficient related to the exciton emission mechanism, yielded α~0.98 for CsP-bI3 QDs and a~1.42 for CsPbI_3_-[SAM]C60 QDs. According to the well-established rule that the coefficient α = 1 is in the case of bound exciton emission and α = 2 is in the case of free exciton emission, the carriers in CsPbI_3_ QDs could be treated as excitons, although they are usually considered to have weak quantum confinement, and the increased α in CsPbI_3_-[SAM]C60 QDs substantiates the fact that there is exciton delocalization [28]. The type II energy-level alignment can result in local electric fields. The electric field can lower the potential barrier at the QD boundaries and further trigger the exciton delocalization. This kind of fullerene-derivative-induced exciton delocalization has also been reported in bulk perovskite film [29]. In addition, theoretical calculations demonstrated that delocalized states can be formed while QD and ligand experience each other’s electric field [30,31]. In brief, the carriers in CsPbI_3_-[SAM]C60 QDs become more dislocated, which is of great benefit to carrier transportation in photovoltaic devices.

**Figure 4:**
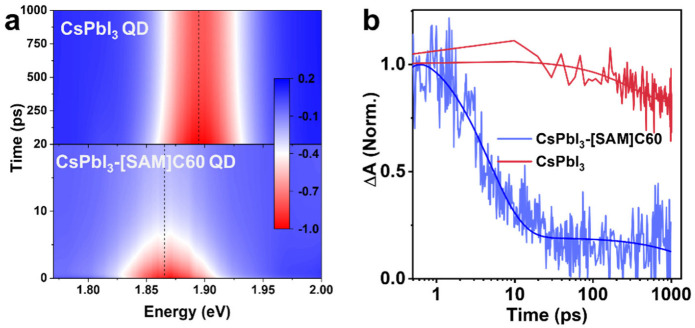
(**a**) Pseudo-color TA maps of CsPbI_3_ QD and CsPbI_3_-[SAM]C60 QD under a 600 nm of excitation wavelength, 16 μJ/cm^2^ of pump fluence. (**b**) Bleaching dynamics for CsPbI_3_ QD and CsPbI_3_-[SAM]C60 QD, respectively.

To directly visualize the electron extraction from C_S_PbI_3_ QD to [SAM]C60, we measured the transient absorption (TA) spectra. Figure 4a shows the pseudo color TA maps of CsPbI_3_ QD and CsPbI_3_-[SAM]C60 QD, respectively. Here, we use a 600 nm pump as the excitation wavelength. The pump intensity is very low to avoid the generation of hot carrier and Auger recombination. The red color regions are the bleaching signals that reflect the transition of electrons at valence band maximum to exciton energy level. As with the absorption spectra, we also observe an obvious redshift in the bleaching peak (from 1.89 eV to 1.87 eV) after the introduction of [SAM]C60. The decay of the bleaching signals is shown in Figure 4b. For CsPbI_3_ QDs, the bleaching signal exhibits very slow dynamics with a lifetime of 501 ps. In contrast, a very fast decay with a lifetime of about 5 ps is observed in CsPbI_3_-[SAM]C60 QDs. The carrier transfer time and the carrier extraction efficiency from CsPbI_3_ QDs to [SAM]C60 can be calculated as 5.05 ps and 99.1%, indicating the excellent carrier extraction ability of [SAM]C60 molecules.

## 3. Conclusions

To summarize, we use [SAM]C60 as the semiconductor ligands of CsPbI_3_ QDs. Compared with other short-chain ligands, the big steric hindrance of the [SAM]C60 molecule keeps the colloidal dispersions stable in solution. Meanwhile, its alignment of the energy levels with CsPbI_3_ QD can form a type II heterojunction, accelerating exciton dislocation and transfer. We observe ultrafast carrier transfer (~5 ps) and highly efficient extraction efficiency (~99.1%) from CsPbI_3_ QDs to [SAM]C60. Our research suggests the great potential of a semiconductor fullerene derivative as the ligand of perovskite QDs and paves the way for efficient CsPbI_3_ QD photovoltaics.

## 4. Materials and Methods

### 4.1. Materials

Oleic acid (OA, 90%), oleylamine (OAm, 70%), octadecene (ODE, technical grade 90%), Cesium carbonate (Cs_2_CO_3_, 99.99%), [SAM]C60, chlorobenzene (CB, 99.8%), were purchased from Sigma-Aldrich (Tokyo, Japan). Lead halide (PbI_2_, 99.99%), methyl acetate (MeOAc, anhydrous 99.5%), n-hexane (99%), and toluene (98%) were purchased from FUJFILM Wako Pure Chemical Corporation (Osaka, Japan).

### 4.2. Colloidal Synthesis of CsPbI_3_ QDs

First, 2.1324 g OA, 0.6 g of Cs_2_CO_3_ and 20 mL ODE were mixed in a 100 mL three-neck flask and degassed at 120 °C for about 40 min. Concurrently, 0.4 g of PbI_2_ and 20 mL ODE were mixed in a 50 mL three-neck flask at 120 °C for 30 min under vacuum. A mixture of 2 mL OA and 2 mL OAm was injected into the PbI_2_ precursor and kept at 120 °C for about 30 min. The mixture was heated to 160 °C under N_2_, then 3.2 mL of the Cs-oleate precursor was immediately injected into the reaction flask, reacted for 5~8 s, and then quickly quenched to room temperature in an ice bath. The colloidal solution was separated into four tubes and 45 mL MeOAc was added, then it was centrifuged at 8500 rpm for 4 min. The precipitated QDs were dispersed in 4 mL of hexane and stored in a refrigerator at 4 °C for 24 h, then centrifuged at 4000 rpm for 2 min. Before being used, the QDs solution was centrifuged at 7000 rpm for 5 min. The supernatant was dried with N_2_ and then dispersed into 50 mg/mL CsPbI_3_ QDs n-hexane solution.

### 4.3. Preparation of CsPbI_3_ QDs with [SAM]C60 (CsPbI3-[SAM]C60 QDs)

Firstly, we added 2 μL [SAM]C60 CB solution (0.02 mmol/mL) to the CsPbI_3_ QDs solution (50 μL, n-hexane). Secondly, the mixture was stirred for 5 min or sonicated for 15 min. Thirdly, the mixture was centrifuged at 4000 rpm for 2 min. Fourthly, the precipitations were redispersed into toluene and centrifuged at 4000 rpm for 2 min. Finally, the QDs were dried with N_2_ then redispersed into 500 μL chlorobenzene or toluene, assisted with a 30 min ultrasound.

### 4.4. Characterization

The phase identification was carried out using a powder X-ray diffraction (XRD, TTR-III, Rigaku Corp., Akishima-shi, Tokyo, Japan). The morphology and crystal structure of the prepared samples were characterized using a transmission electron microscopy (JEM-2100F, JEOL Ltd., Akishima, Tokyo, Japan). UV-vis absorption spectra were measured with a spectrophotometer (HITACHI, U-3900H, Minato-ku, Tokyo,, Japan). Photoluminescence Quantum Yield (PLQY) and steady Photoluminescence (PL) was measured with the Absolute PLQY Measurement System (from C11347 Hamamatsu, Hamamatsu City, Japan) at an excitation power of 0.1 mW. Transient PL. PL are all recorded with the PL system from TOKYO INSTRUMENT, INC. A 473 nm pulsed diode laser (pulse width 90 ps, repetition up to 100 MHz, peak power 4 mW) was used as the excitation source. An adjustable neutral density filter was adopted to adjust the excitation intensity. The PL detection was used with a PMT together with a TCSPC module. Transient absorption (TA) measurements were performed using an fs TA setup. The laser source was a titanium/sapphire laser (CPA-2010, Clark-MXR Inc., Huron River, MI, USA) with a wavelength of 775 nm, a repetition rate of 1 KHz, and a pulse width of 120 fs. The light was separated into two parts. One part was used to stimulate a sapphire plate to generate white light for the probe beam. The other part was used to pump an optical parametric amplifier (OPA) (a TOAPS from Quantronix) to generate light pulses with a wavelength tunable from 290 nm to 3 μm. It was used as a pump light to excite the sample. In this study, a pump light with a wavelength of 600 nm was used to excite the QDs.

## Figures and Tables

**Table 1:** Parameters fitted from absorption spectrums with Elliott formula.

	CsPbI_3_	CsPbI_3_-[SAM]C60
E_g_ (eV)	1.92	1.90
E_b_ (meV)	25	7
T (meV)	32	32
